# Examination of the roles and capacities of duty bearers responsible for protecting the human rights to adequate food, nutritional health and wellbeing in Ugandan children’s homes

**DOI:** 10.1186/s12914-018-0156-4

**Published:** 2018-04-17

**Authors:** Monica Olafsen, Archangel Byaruhanga Rukooko, Per Ole Iversen, Bård A. Andreassen

**Affiliations:** 1 Department of Nutrition, Institute of Basic Medical Sciences, University of Oslo, P.O. Box 1046 Blindern, 0317 Oslo, Norway; 2 School of Liberal and Performing Arts, Makerere University, Kampala, Uganda; 3 Division of Human Nutrition, Faculty of Medicine and Health Sciences, Stellenbosch University, Cape Town, South Africa; 4 Norwegian Centre for Human Rights, Faculty of Law, University of Oslo, Oslo, Norway

**Keywords:** Children’s home, Duty bearer, Human right to adequate food, Obligations, Role and capacity analysis

## Abstract

**Background:**

The majority of Ugandan children face vulnerability and malnutrition. As a State Party to international human rights treaties, Uganda has legal obligations of guaranteeing the fundamental rights and the best interest of the nation’s children. Despite being protected under international and national law, Uganda is not providing adequate child protection, including safeguarding children’s food security. Numerous privately owned and unregulated children’s homes face this problem. The overall aim of the study was to examine to what extent children’s homes’ operations are consistent with the right to adequate food, nutritional health and wellbeing of children.

**Methods:**

We performed a qualitative role- and capacity analysis of duty bearers with human rights duties towards children living in children’s homes. We studied three groups of duty bearers: caretakers working in private children’s homes, State actors working in government and its institutions, and non-State actors working in civil society organizations. A human rights based approach guided all aspects of the study. An analysis of the roles, performance and capacities of duty bearers was employed, with individual face-to-face structured qualitative in-depth interviews, self-administered structured questionnaires, and a structured observational study, as well as a desk review of relevant literature.

**Results:**

The State of Uganda’s efforts to respect and realize its obligations towards children living in children’s homes is inadequate. There are numerous capacity gaps among the duty bearers, and the concepts of human rights and the best interest of the child are not well understood among the duty bearers.

**Conclusion:**

The efforts of the State of Uganda to realize its human rights obligations towards children in children’s homes are lacking in important areas. Hence the State does not fulfill its minimum obligations under the International Covenant on Economic, Social and Cultural Rights to ensure all children freedom from hunger. There is a need for capacity development at all levels in the Ugandan state and the international society to delimit capacity gaps in order to realize these human rights’ obligations.

**Electronic supplementary material:**

The online version of this article (10.1186/s12914-018-0156-4) contains supplementary material, which is available to authorized users.

## Background

Uganda is rich in natural resources and well suited for agricultural production. However, there is prevalent vulnerability to food insecurity and deprivation of the human right to adequate food at the local level, in part due to seasonal food crop variations and uneven food accessibility and distribution [1]. Food taboos and food distribution at household level are culturally determined along age- and gender lines. In effect, children, particularly the girls, are vulnerable to food shortages and inadequacy [2].

Ugandan children face high prevalence of poverty [3], disease and malnutrition [4], affecting their survival, health, development, wellbeing, future economic performance, and consequently the intergenerational detrimental cycle of malnutrition and poverty [5, 6]. Children comprise more than half of the Ugandan population [4], and 96% have been classified as having some degree of vulnerability, with 51% being moderately or critically vulnerable [7]. Moreover, children not living with their biological parents are of concern as they are at risk of exploitation, neglect and/or violence [8]. Nineteen percent of Ugandan children are living with neither of their biological parents and 32,000 children are living without an adult caregiver [4, 9].

An ‘approved home’ is a governmental or non-governmental institution approved by the Minister of Children’s Affairs under the Ministry of Gender, Labour and Social Development to provide alternative care for children below the age of 18 years with a ‘care order’ of court. In order for the children’s homes to assume the legal responsibility for the children in their care, the children need a ‘care order’ issued by the police or local government within the first 24 h of their arrival at the children’s home. While the children’s homes actually should be ‘approved homes’ with a license received from the Minister of Children’s Affairs under the Ministry of Gender, Labour and Social Development, this has been received only by very few children’s homes. Nevertheless, even without this license, children’s homes may assume the legal responsibility for vulnerable children through a ‘care order’. The ‘approved homes’ are subjected to regulations under the Approved Homes Regulations under sections 66 and 110 of the Children Act, Cap 59 [10, 11]. In spite of the need for children’s homes to resolve acute situations for some children’s wellbeing, growing up in children’s homes may have damaging effects on children’s physical, psychological, social and cognitive development; whereas families often provide the best environment for children’s growth and development [12]. The Government’s legal and policy frameworks emphasize reducing the rates of institutionalization by supporting family reintegration, family and community-based empowerment and care, and economic strengthening [13]. In 2015, there existed more than 850 private non-approved children’s homes in Uganda (UNICEF Consultant Mark Riley, personal communication), and in 2012 the numbers of children living permanently in children’s homes exceeded 57,000. Approximately 50,000 of these were separated from their families, and more than half were admitted without a ‘care order’, thus being there illegally [14].

There is limited research on the situation of vulnerable children receiving care and the state of the children’s homes in Uganda. According to investigations, most children’s homes do not comply fully with human rights standards, with negative effects on living conditions and the provision of care [14–18]. This includes limited healthcare provisions and inadequate access to clean drinking water, a very limited diet, and malnourished-looking children. As most children’s homes are not subject to adequate Government control, the potential for disregard of the human rights and of the best interest of the child is imminent. The Approved Home Regulations [11] guide the operation of approved children’s homes. Yet these guidelines do not specify food and nutrition standards.

The overall aim guiding the study was to examine the performance of the State of Uganda in meeting its legal obligations accruing from international and domestic human rights law to respect, protect and fulfill the rights relevant to obtain good nutritional health and wellbeing of children receiving care in children’s homes in Uganda. Our study has two parts: 1) To examine the institutional structures and legal, policy and program framework supporting the right to adequate food, nutritional health and wellbeing of vulnerable children receiving care in children’s homes, and the consequent duties of duty bearers. 2) To perform a qualitative role and capacity analysis of duty bearers with duties towards children receiving care in children’s homes. To this end, we first performed a desk review of the institutional structures and legal, policy and program framework supporting the right to adequate food, nutritional health and wellbeing of the children, the consequent roles and duties of duty bearers, and Uganda’s overall compliance with relevant human rights obligations. Based on the findings from the desk review we developed adequate research tools and then performed a qualitative capacity analysis investigating the subjective perceptions of selected duty bearers concerning their duties towards the children. Finally, we analyzed the findings, based on both the desk review and the investigations of duty bearers. This analysis was structured according to the State’s obligations to respect, protect, and fulfill human rights as the overall aim of the study, and to the five elements of capacity linked to the following five research questions:

1. To what extent are legal, administrative, social and cultural factors in the responsible institutions, constraining duty bearers to perform their duties to realize the human right to adequate food to obtain good nutritional health and wellbeing of children in children’s homes in Uganda?
2. To what extent do duty bearers recognize their roles in terms of meeting their above mentioned duties?
3. To what extent do duty bearers have the managerial, economic, and organizational capacity to fulfill their duties to promote, support and/or implement the human right to adequate food and nutritional health in children’s homes?
4. To what extent do duty bearers communicate effectively in order to fulfill their duties to uphold the rights of the children?
5. To what extent do duty bearers have the capacity for making decisions to implement the right to adequate food and nutritious health?

## Methods

### Study participants

A duty bearer in the present study was a person with duties towards protecting and realizing the rights of children, with particular emphasis on those responsible for orphans and those most vulnerable with need for care. The three groups of duty bearers comprised (i) caretakers working in the five randomly selected privately owned children’s homes in the Kampala ‘extra region’ [16], (ii) State employees working in Government departments and its institutions, and (iii) non-State employees working in national and international civil society organizations.

Participants were purposefully selected, based on the performed ‘role and responsibility analysis’. Table 1 shows the number of study participants, as well as the method of participation in investigation. The research team (consisting of the first author and one research assistant) performed in-debt interviews with four respondents in each of the five children’s homes. At central Government level the focus was on duty bearers working in the Department of Youth and Children Affairs of the Ministry of Gender, Labour and Social Development as the lead State agency responsible for the welfare of all children, including those in children’s homes. These State actors are in a position to advocate for and ensure legislation and strategy implementation. At local Government level, the focus was on State actors responsible for strategy implementation. At civil society level, the focus was on duty bearers emphasizing the national framework and strategies in their work, and those emphasizing the rights of the child. Eligible key informants were from other institutions that did not have any apparent duties towards ensuring the rights of children receiving care in children’s homes. They were purposively selected based on their knowledge of technical information from their respective fields of expertise that we found significant and valuable to increase the understanding of the Ugandan society of relevance to the study.

**Table 1:** Study participants

	Interview guides		
	In-depth interviews	Self-administered questionnaires	Customized interviews
Caretakers (five children’s homes)	*n* = 20	*n* = 26	
State actors (Government and its institutions):	*n* = 9	*n* = 2	
Department of Children Affairs			
Local Governments			
Uganda Human Rights Commission			
National Council for Children			
Non-State actors (civil society organizations):	*n* = 4	*n* = 11	
Uganda Child Rights NGO Network			
UNICEF Uganda			
Uganda Women’s Effort to Save Orphans			
Raising Voices			
ANPPCAN Uganda Chapter			
Key informants:			*n* = 9
- National Coordinator Coordination Office for the National Child Protection Working Group; Ministry of Gender, Labour and Social Development			
- Assistant Commissioner Department for Human Resource Development, Planning and Quality Control; Naguru Police			
- Program Manager Department for Research, Communication and Advocacy; ANPPCAN Uganda Chapter			
- Student Human Rights Activist; Makerere University			
- Director Human Rights Centre Uganda; United Nations Special Rapporteur on the situation of Human Rights Defenders. Former Chairperson of the Uganda Human Rights Commission (1996–2008)			
- Director Reev Consult International; Professor in Social Science Makerere University, World Bank consultant, Managing Consultant on research on poverty reduction			
- Makerere Research Institute			
- Director of Complaints, Investigations and Legal Services; Uganda Human Rights Commission			
- Founder Alternative Care Initiative; Alternative Care Consultant Ministry of Gender, Labour and Social Development			

### Theoretical frameworks

Three theoretical frameworks guided the present study; the international human rights framework, the normative UNICEF conceptual framework [19], and Kent’s conceptual framework of nested rings of responsibility [20, 21].

As part of the international human rights framework, the focus of the study has been on the human rights based approach, and on human rights standards and principles in the Covenant on Economic, Social and Cultural Rights (CESCR) [22] and the Convention on the Rights of the Child [23], as well as their respective General Comments made by the monitoring bodies of these conventions. Ratification by the State of Uganda [24, 25], as the principal duty bearer, established legal entitlements of protection for the best interest of the child and the human rights of Ugandan children to adequate food, nutritional health, and wellbeing. “The right to adequate food” has been applied as elaborated in General Comment 12 in Article 11 of the CESCR. This right implies available, safe and culturally acceptable food, meeting the child’s dietary needs in quality and quantity. Further, it implies economical and physical sustainable accessibility of food. Thus, as Party to these treaties [22, 23], the principal obligation of the State of Uganda is to undertake steps, to the maximum extent of available resources, without any discrimination, with a view to achieve progressively the full realization of the right to adequate food and related rights. With reference to human rights law, Uganda is required to domesticate treaties that is, making it part of national legislation and policies. Uganda is further obliged to take whatever steps necessary to ensure for every child access to the minimum essential food. Such food shall, at the very minimum, ensure children’s freedom from hunger. Table 1 in Additional file 1 provides more information, and in Table 2 in Additional file 1 we present international treaty provisions supporting the rights of the child. Provisions on the best interest of the child, in accordance with the views of the child, as established in the Convention on the Rights of the Child [23], are presented in Table 3 in Additional file 1. The standards of the international human rights framework are further protected in Uganda’s national legal and policy framework, with attention towards vulnerable children, deinstitutionalization, and family preservation and reunification. National legislation of particular interest in this study was the Constitution [26], the Children Act [10], and Uganda’s food related provisions presented in Table 4 in Additional file 1.

**Fig. 1:**
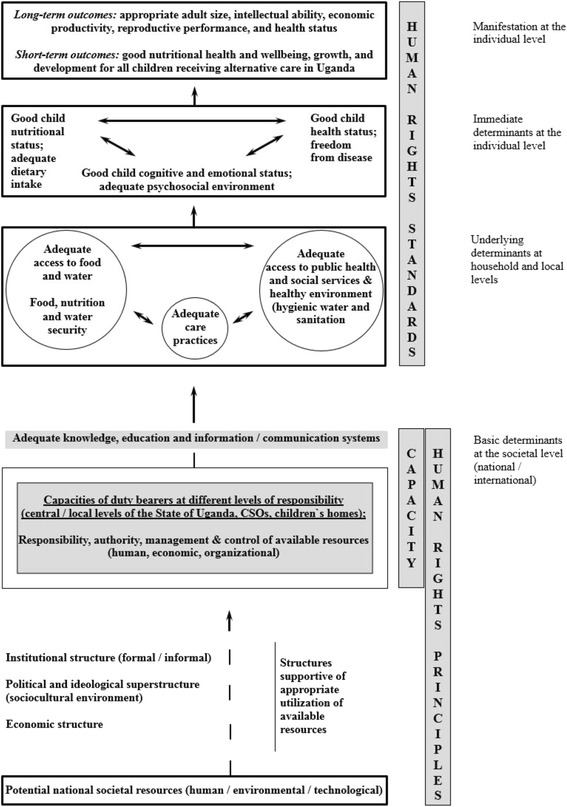
The human rights normative conceptual framework for identifying the determinants for good child nutritional health and wellbeing, growth and development. Inspired by different variations of the framework as adapted from [5, 6, 19, 29, 38–42]

The normative UNICEF conceptual framework reflects that the nutritional status of a child, and thus child wellbeing and development, is an outcome of complex biological and societal determinants and processes, and illustrates how care must be taken at all three levels of determinants to prevent structural conditions for the creation or maintenance of malnutrition. Further it provides a theoretical framework for a role and capacity analysis and can be used for the identification of State obligations, and thus for strengthening the capacities of duty bearers in addressing the various determinants for the realization of good child nutrition. Figure 1 shows the framework adapted to the present study. It illustrates how resources and structures of the society influence on the child’s access to food, care and health services, and thus on their nutritional health and wellbeing. It further takes into account human rights standards and principles, as well as the capacities of duty bearers. Governance and the utilization of resources depend on how problems are understood and the values and priorities of those who control them. This in turn is influenced by education and information.

**Fig. 2:**
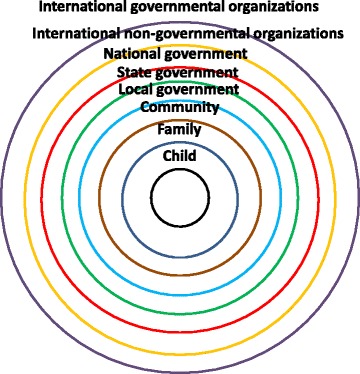
The conceptual framework of nested rings of responsibilities, modified after Kent [21]

Kent’s conceptual framework of nested rings of responsibility (Fig. 2) illustrates governance and how various duty bearers are located at different levels of proximity to the child as rights holder. By realizing the rights of duty bearers, they will thus be enabled to meet their duties towards other right holders. If duty bearers at a certain level are failing to meet their duties, duty bearers at a more distant level should not replace the closer ones, but strengthen and empower them so that they can meet their duties. Under international human rights law, the State is the principal duty bearer with legal obligations of progressively realizing human rights. However, the realization of the human rights of children are the shared responsibility between various State and non-State actors within the society; non-State actors are obliged to respect human rights as these treatises are part of the legal framework of the country. While these duty bearers do not have the capacity to sign treaties, they still have a duty to create enabling environments for human rights realization.

### Qualitative human rights based analysis

In the present study we performed a causality and performance analysis, a role and responsibility analysis, and a capacity analysis [19, 27, 28], at the basic level of the normative UNICEF conceptual framework.

A causality and performance analysis is the basis for a role-, responsibility- and capacity analysis. It consisted of a desk review, namely a document analysis to examine the current situation of children living in Uganda’s children’s homes, the country’s legal and policy framework for vulnerable children, as well as the legal obligations of duty bearers. In addition we conducted a simple structured observation analysis in the five children’s homes. Semi-structured qualitative interviews were carried out with key informants, adding technical information from their fields of expertise (Additional files 2, 3 and 4).

The role and responsibility analysis was guided by the concept of Kent’s nested rings of responsibility together with the findings from the causality and performance analysis. It involved the identification of the three groups of responsible duty bearers at different levels of proximity to the child, as well as the establishment of their specific roles and duties towards children living in children’s homes. Its outcome was a list of eligible duty bearers relevant for purposeful recruitment for investigation in the capacity analysis.

The capacity analysis involved the investigation of the capacities of the selected three groups of duty bearers for ensuring the rights of vulnerable children in children’s homes. For the capacity analysis we developed qualitative research tools, the foundation of which was the human rights framework, the human rights based approach, the normative UNICEF conceptual framework, Kent’s nested layers of responsibilities, and the role and capacity analysis framework including the outcome of the abovementioned analysis. The following five elements of capacity were investigated:

- Capacity I: Authority to make decisions and to take action;
- Capacity II: Duty bearers’ motivation, commitment and acceptance of duty;
- Capacity III: Availability, access and control over relevant economic, human and organizational resources to enable decision-making and action;
- Capacity IV: Resources and capabilities for effective communication;
- Capacity V: Capabilities for informed and rational decision-making and learning from experience.

Individual face-to-face qualitative in-depth interviews were the main method of obtaining data, in addition to self-administrated objectives-based structured questionnaires. The emphasis was on the capacities necessary to enable duty bearers to fulfill their duties, and to search for information, knowledge and perceptions held by them. Issues of relevance included the situation and human rights of children living in the children’s homes; push and pull factors driving the situation of orphans and other vulnerable children and their institutionalization; factors threatening the traditional extended family social protection and security mechanisms; and actual efforts towards the implementation of the national alternative care framework [13]. In addition we developed a research tool to guide a simple structured observation analysis in the five children’s homes, as well as individualized semi-structured qualitative interview guides for key informants.

### Qualitative data processing and analysis

The causality and performance was compared with the study findings to evaluate the representativeness of these findings. The causality analysis contributed to the fulfillment of the aim to examine the framework supporting the right to adequate food, nutritional health and wellbeing of children living in children’s homes, and the consequent duties of duty bearers. The performance analysis contributed to the fulfillment of the overall aim guiding the study, namely to examine how the State is meeting its obligations to respect, protect and fulfill these rights.

The legal duties of duty bearers, as well as Kent’s conceptual framework of nested rings of responsibility, guided the process of identifying specific duty bearers as well as the establishment of their specific roles, duties, performances, and capacities in meeting their duties as regards implementing the human rights of vulnerable children in Uganda. The role and responsibility analysis contributed to perform a qualitative role and capacity analysis of duty bearers with duties towards children receiving care in children’s homes.

The analysis of the five elements of capacity involved assessing the ability of duty bearers in meeting their duties and undertake actions for human rights realization. This implied their performance in accordance with established norms and standards taking into account their efforts and achieved results and the resources available. This analysis was grounded in the initial desk review, namely the human rights based causality, role, and performance analysis, with the purpose to support or shed light on findings. The capacity analysis was applied by way of a qualitative assessment of in depth interviews, self-administrated questionnaires, and the observational studies in the children’s homes. To direct the analysis and discussion of findings, three study tools were developed and the questions were organized under the headlines of the five elements of capacity and sub-headlines of the international human rights principles and standards, as well as linking these to the research questions and normative indicators. The objective was to determine capacity gaps in meeting duties, implying how duty bearers’ performance deviates from the ideal norm. The examination of capacity gaps was seeking to find out why duty bearers were not fulfilling their duties. Any revealed gaps may need to be resolved before rights can be enjoyed, and is thus important for the identification of areas in need of capacity development of duty bearers to address the various conditions for the realization of good child nutrition. The capacity analysis contributed to the fulfillment of part two of the study aim, and to the fulfillment of the overall aim guiding the study.

To answer the five research questions, the findings of the total human rights analysis were finally analyzed and discussed under the headlines of the five elements of capacity and the international human rights obligations to respect, protect and fulfill the rights relevant to obtain good nutritional health and wellbeing. The findings were compared between the three groups of respondents.

The qualitative tools of the capacity analysis were structured and objectives-based, and questions were developed and organized in a matrix in accordance with the five elements of capacity and the international human rights standards and principles, as well as the research questions and normative indicators. Each individual’s answer to every interview question was then pasted into this matrix in one single document, and further arranged in accordance with the groups of respondents.

A systematic content analysis was applied to the investigation of all study data linked with indicators, namely of documents considered essential and valuable for the realization of the aim of the study, and a role, performance and capacity analysis as part of a case study approach. The content analysis was a continuous process before, during and after data collection. Focus was on capacity gaps and the need for capacity development of relevant duty bearers, and the gathered data was compared with the normative indicators for performance and capacity.

## Results

### The five elements of capacity necessary for duty bearers to meet their obligations

The following provides findings from the human rights analysis, where findings from the capacity analysis of duty bearers at three different levels of responsibility has been compared with findings from the causality and performance analysis.

Capacity I involves the authority of duty bearers to make decisions and to take action. We made three observations:

1. All respondents perceived they have legal and/or moral authority and legitimacy to act to reduce the suffering of children. However, there were capacity gaps: i) Lack of legal authority and mandate is not considered a constraint to act. This includes the inconsistence of following the Government strategies of family preservation and deinstitutionalization of children, and as such to recruit children to live in children’s homes. ii) There is a widespread approach of benevolent behavior based on needs and charity, as opposed to the human rights based approach [28], towards providing care for vulnerable children as a duty.
2. All respondents have awareness of the Ministry of Gender, Labour and Social Development as the lead line ministry responsible to realize the human rights of vulnerable children. However, a capacity gap is the lack of awareness of the mandate and authority of the Ministry of Agriculture as well as the Ministry of Health, as the line ministries responsible for ensuring the right to food and food security policy.
3. Other capacity gaps hindering duty bearers to fulfill their duties include: i) lack of human and financial resources and ii) non-compliance with the human rights based approach for good governance. There is excessive bureaucracy, hierarchy and corruption at all levels of the Ugandan society. There is misuse of authority, and inadequate structures of accountability. As such, there are social, legal-administrative, and cultural barriers to overcome.

Capacity II involves duty bearers’ motivation, commitment and acceptance of duty. We made two observations:

1. Uganda recognizes and acknowledges that it should act, through being a State Party to all relevant international and African human rights instruments. Uganda further acknowledges that it should act towards vulnerable children, its on-time submission of initial and periodic reports to the United Nations Committee on the Rights of the Child, and its oversight role. However, there were capacity gaps hindering duty bearers to fulfill their duties: i) the inadequate domestication of the ratified human rights instruments into national legislation indicates a lack of State commitment to and compliance with human rights standards. The human right to adequate food is not included in the legally binding Chapter 4 of the Constitution of the Republic of Uganda [29], and is as such interpreted as mere non-justiciable political objectives or guiding principles. While the work with the Food and Nutrition Policy, Strategy and Bill [30–32] was initiated more than two decades ago, it was still awaiting Cabinet approval for Parliament legislation. Among the consequences is the lack of instituting the Uganda Food and Nutrition Council, the lack of provision of adequate funds and other resources, as well as lack of policy implementation. As regards the human right principle of the best interest of the child, there has been prolonged time for the enactment of the revised Children Act [33], the Alternative Care Framework [13], and the Approved Home Regulations [11] developed by the Ministry of Gender, Labour and Social Development. This Ministry does apply the two latter frameworks, however without them being adopted as legal provisions. This adversely affects provision of funds and resources, and hence, the implementation of the policies. ii) There is a widespread lack of awareness of and adherence to the concept of the human rights based approach for good governance among duty bearers. iii) There have been delays in the State submission of reports to the respective United Nations treaty monitoring agencies, the Ministry of Gender, Labour and Social Development being one exception. iv) The State’s total public social expenditures are being smaller than funds allocated to infrastructure, public administration, and military sectors, and in addition, the State does not have any social assistant grant scheme for poor and vulnerable children [34–36].
2. All study respondents at all levels of responsibility had knowledge of the adverse health effects of an inadequate food intake for children. However, there were capacity gaps: i) lack of available food and thus lack of food provision for children, in particular lack of provision of nutritious food. ii) A charity-based approach towards caring for vulnerable children was widespread among respondents and iii), there was low awareness and acceptance of human rights and the rights of the child. Human rights were by many respondents only considered essential within the setting of an income generating activity.

Regarding capacity III, it concerns proper management of economic, human and organizational resources for meeting their duties towards vulnerable children. We made three observations:

1. Resource limitations affected all the capacities and research questions in the present study.
2. Uganda has enough available food resources to feed all its citizens. However, capacity gaps were hindering duty bearers to fulfill their duties: i) the country has an inadequate food distribution system, leading to food insecurity. ii) There is lack of State protection of ancestral land of individuals and groups, as well as other natural resources.
3. The national budget is allocated towards infrastructure, such as the building and maintenance of roads, which is important for increasing food accessibility at regional and local levels. However, capacity gaps included: i) the State prioritizes budget allocations towards national security and military, while relatively small allocations go towards programs for food security for vulnerable children managed by the Ministry of Agriculture, the Ministry of Health, as well as the Ministry of Gender, Labour and Social Development, the least funded ministries in Uganda. There is no nutrition budget, and public spending on agriculture and health is low. Duty bearers in these ministries are dependent on benevolence of non-State organizations and other countries to perform their duties. The National Council for Children and the Uganda Human Rights Commission are dependent on external donor funding. This may be a potential threat to their independence. ii) All study respondents described financial resource mobilization as a time consuming activity, adversely affecting the realization of their duties.

Capacity IV involves duty bearers’ resources and capabilities for effective communication. We made three observations:

1. Quality information about human rights of the child, children’s homes, vulnerable children, and food adequacy was readily available at the internet in the English language. However, capacity gaps included widespread lack of access to internet and information was not available in most local languages.
2. While good communication-coordination mechanisms existed between different levels of Government and the civil society, lack of resources adversely affected accessibility, namely the sharing and dissemination of relevant information as well as the development of the communication capacity of duty bearers.
3. The State raises awareness and does education activities towards the population. However, capacity gaps included: i) inadequate popularizing through oral distribution of information as well as media coverage including rural radio. ii) Inadequate advocacy efforts to raise the prestige of vulnerable children, as evident by the continued insufficiency of Government resource allocation towards the Ministry of Gender, Labour and Social Development.

With regard to capacity V we asked if the duty bearers had the capacity for making decisions to meet their duties towards vulnerable children. We made two observations:

1. Most relevant legal and policy frameworks in Uganda are human rights based. However, there were capacity gaps: i) There was generally low awareness and acceptance of the concept of human rights, including the rights of the child, comprising the important human rights principles of the right to be informed, to have a voice in matters of concern, and to participation and involvement. Hence, there was general lack of understanding of meaningful child participation. ii) With reference to the concept of human rights based approach for good governance, Uganda has put in place several measures to raise awareness of and fight corruption, as well as to build ethics and integrity in the society.
2. Regular demographic monitoring and evaluation efforts of vulnerable groups in general were conducted at the national level, as well as by the Ministry of Gender, Labour and Social Development. However, we observed inconsistencies between different monitoring and evaluation establishments, which may create unclear statistics. One example of this is UNICEF’s orphan statistics which coincides with the Ugandan national statistics, but differs from other European statistics.

### The State’s obligations to respect, protect, and fulfil the human rights relevant to obtain good nutritional health and wellbeing of vulnerable children in children’s homes

The findings from the capacity-, the causality- and the performance analysis were next examined in view of the legal international human rights obligations of duty bearers to respect, protect and fulfill the rights relevant to obtain good nutritional health and wellbeing of children living in children’s homes, as follows:

Does the State of Uganda meet its obligation to respect the human rights relevant to obtain good nutritional health and wellbeing of vulnerable children living in children’s homes in Uganda? The State does meet this obligation through the following: 1. The State is supportive of and encourages the exercise of optimal food practices, deinstitutionalization and family based care, participation in transparent decision-making processes, and the applicable legislation and strategy recommendations. 2. The content of State-sponsored information, education and communication messages is technically sound and based on international and national guidelines. 3. The State listens to and respects the views and opinions of vulnerable children, relevant stakeholders, and the media.

Does the State of Uganda meet its obligation to protect the human rights relevant to obtain good nutritional health and wellbeing of vulnerable children living in children’s homes in Uganda? The State meets this obligation to varying degrees. 1. The State has ratified all relevant human rights instruments. However, the human right to adequate food is not incorporated into relevant national legislation. 2. Many lack the inclusion of goals and benchmarks, while most lack the allocation of adequate resources and are thus not being effectively implemented, monitored and evaluated, and enforced.

Does the State of Uganda meet its obligation to fulfill (facilitate) the human rights relevant to obtain good nutritional health and wellbeing of vulnerable children living in children’s homes in Uganda? The State meets this obligation to varying degrees. 1. The State has created mechanisms and institutions at different levels of Government responsible for supervision. This includes an institutional framework for multilevel, intersectoral and multidisciplinary communication, cooperation and coordination, and a national monitoring and evaluation and feedback mechanism. As regards reporting to and the follow up on international monitoring and evaluation mechanisms, implementation is however variable. 2. The State is not meeting its facilitation obligation of ensuring the following: First, ensuring adequate deinstitutionalization and family based care through the strengthening of the extended family social protection mechanism, facilitation of parental job creations or other income or food generating activities, and the retention of children in school. Second, ensuring accountable and empowered duty bearers at all levels of the society. Finally, ensuring the respect of human rights, the rule of law, and the human dignity of children.

Does the State of Uganda meet its obligations to fulfill (provide) the human rights relevant to obtain good nutritional health and wellbeing of vulnerable children living in children’s homes in Uganda? The State does not meet this obligation, in particular regarding needy children vulnerable to malnutrition in children’s homes.

Does the State of Uganda meet its obligations to fulfill (promote) the human rights relevant to obtain good nutritional health and wellbeing of vulnerable children living in children’s homes in Uganda? The State meets this obligation to varying degrees. 1. State actors do encourage deinstitutionalization and family based care for vulnerable children. 2. The State does, however, not promote national food and nutrition initiatives and measures encouraging optimal food practices.

## Discussion

The five elements of capacity necessary for duty bearers to meet their obligations are discussed below.

With regard to Capacity I, in order to meet their human rights duties, duty bearers need the capacity of mandate and authority to be able to take action. However, formal and informal rules, norms and power relations, may influence such facilitation of authority. Engh [37] argues that once the State obligations are identified, there is a need to define mandates and authority that translate such obligations into relevant policies.

While most duty bearers reported that they have legal and/or moral legitimacy to act in accordance with a duty to promote and/or realize the rights of children living in children’s homes, they reported of social, legal-administrative, and cultural barriers to overcome. While most respondents were aware of the mandate and authority of the Ministry of Gender, Labour and Social Development as the lead line ministry responsible for policy implementation relevant for vulnerable children, the mandate of the Ministry of Agriculture, Animal Industry, and Fisheries and the Ministry of Health as the line ministries responsible for ensuring the human right to food and food security policy, was not recognized. The mandate of the society, including parents and children’s homes, was mostly recognized as a moral duty to care for vulnerable children and not a legal duty. The respondents did not consider the lack of legal authority and mandate, nor the inconsistence of following the Government strategies, as a constraint for duty bearers to take action as regards their human rights duties towards vulnerable children. As such, there is need for enactment of the Food and Nutrition Bill [32] to establish a home for its Council to be able to efficiently carry out its oversight and capacity development mandate. Further, there is need for capacity development as regards the legal and moral responsibility of parents and families to care for their children as well as the strategy of deinstitutionalization as outlined in continuum of care of the revised Children Act, the Alternative Care Framework [13], and the Approved Home Regulations [11], which is not yet enacted, as well as the National Orphans and other Vulnerable Children Policy, and Programme Plan. The enactment, implementation, and capacity development as regards this framework is necessary to realize the concept of the best interest of the child and the human rights of Ugandan children to adequate food, nutritional health, and wellbeing [22, 23]. This would contribute to a change of focus, from a charity approach towards a human rights based approach, as regards the realization of the right to food, health and care for vulnerable children as a duty. The basic needs and charity approaches to development emphasize finding resources to alleviate the manifestation or immediate causes of problems and relieving suffering. These approaches recognize a moral responsibility of rich towards poor, and individuals are seen as victims or objects that deserve assistance. In contrast to the human rights based approach, there is no obligation to meet needs through focusing on structural causes, manifestations and injustices nor the redistribution of existing resources, and as such needs for adequate food, healthcare and care are met only when resources are available.

As regards decentralization and authority of local Governments, the respondents considered there was adequate mandate and administrative and financial authority to carry out policies relevant for vulnerable children. There was a lack of sustained commitment as reflected in excessive bureaucracy and corruption and lack of human and financial resources at all levels of the society. As such, the structure of accountability, an essential part of the human rights based approach for good governance, was not adequately implemented to ensure that duties were performed in accordance with obligations and that duty bearers were accountable to the proper authorities. With the exception of civil society organizations, duty bearers at lower levels of the hierarchy often felt that they were restricted in term of influence on decision-making.

In terms of capacity II, acceptance of legal and/or moral responsibility involves duty bearers’ motivation to implement measures towards the enjoyment of a human right, and the internalization, commitment, and leadership taken in this regard. From a human rights perspective it is important to distinguish political will from capacity, i.e. where the inability to perform a duty is interpreted as lack of capacity to do so as compared to unwillingness to comply.

As a State Party to all relevant international and African human rights instrument as well as through its legal and policy framework, Uganda recognizes and acknowledges that it should do something about the nutritional health and wellbeing of its population and in particular its vulnerable groups such as children. These issues indicate acceptance of obligations on the part of the State. Ratification of human rights instruments is however a limited indicator of political commitment as it is a one-time event. The lack of legislating the human right to adequate food into the legally binding Chapter 4 of the Constitution of the Republic of Uganda [26] and the Food and Nutrition Bill [32], the lack of instituting the Uganda Food and Nutrition Council, the lack of provision of adequate funds and other resources to realize the human right to adequate food, as well as the lack of policy implementation, indicate a lack of State acceptance as regards meeting its legal obligations of the right to adequate food. The prolonged time for the enactment of the revised Children Act [33], the Alternative Care Framework [13], and the Approved Home Regulations [11] adversely affects provision of funds and resources to the Ministry of Gender, Labour and Social Development, and hence, the implementation of the policies of relevance to realize the principle of the best interest of the child. Furthermore, the prolonged time for the State submission of initial and periodic reports according to schedule to the respective United Nations treaty monitoring agencies, indicates a lack of commitment towards international obligations. The State does not have a social assistant grant scheme for poor and vulnerable children. Political leadership has not taken practical steps towards allocating national resources towards realizing the human right to nutritional health and wellbeing of vulnerable children in children’s homes. However, The Ministry of Gender, Labour and Social Development, the most underfunded ministry in Uganda, showed acceptance towards its legal obligations relevant for vulnerable children through its policy framework and its work in this regard.

The duty bearers recognized elements of the human right to adequate food, however lack of capacities limited the acceptance towards the vulnerable children in the children’s homes. While children’s home respondents should acknowledge their special role in providing nutritional health and wellbeing to children in their care, the lack of available resources was considered a valid reason for the lack of available nutritious food. Duty bearers, particularly in the children’s homes, believed in short term food and other necessity handouts. None of the duty bearers recognized how this was opposed to the human rights based approach and the human right of the child to adequate food [22, 23]. Some duty bearers distinguished between the commitments to their occupational legal human rights duties as compared to their private moral duties, i.e. they did not necessarily accept the concept of human rights of the child and as such saw human rights work only as an income generating activity.

There was generally low acceptance and internalization of basic human rights and the rights of the child in the Ugandan society. Since placing children in the children’s homes is a direct choice of child protection strategy of parents and civil society organizations, which is contradictive to the Government policy of child protection, there is a clear violation of the principle of the best interest of the child and the legal rights of the child to parental care [22, 23]. Moreover, as there was a deliberate choice made by duty bearers in the children’s homes to spend the limited available resources on institutional rather than on parental care for the children. As such, duty bearers at all levels were not responsive to vulnerable children and their needs. As most children’s homes’ staff operated without adequate knowledge of the human rights and the best interest of the child, and were not following the Ministry of Gender, Labour and Social Development approach of deinstitutionalization, there were misuse of authority and lack of commitment and subsequent violation of the right of many children to be cared for by their parents.

Capacity III involves having access to and control over necessary economic, human, and organizational resources required to address a problem and meet a duty. Uganda has enough available food resources to feed all its citizens adequately in terms of quantitative and qualitative adequacy, safety and cultural food acceptance, including malnourished groups. Nevertheless, food insecurity at regional, local and household levels is extensive due to amongst others an inadequate food distribution system, and hunger and malnutrition is periodically widespread in the country. Furthermore, the State does not ensure adequate food storage as a backup for times of disasters. As such, the State has not fulfilled its obligation under the CESCR [22] nor under its Constitution [26]. Article 11.2(a) of the Covenant [22] states that the States Parties, “recognizing the *fundamental right of everyone to be free from hunger*, shall take, individually and through international co-operation, the measures, including specific programmes, which are needed to improve methods of *distribution of food* by making full use of technical and scientific knowledge”. Objective XIV (b) of the Ugandan Constitution [26], interpreted only as non-justiciable political objectives or guiding principles, states that the State shall ensure that all Ugandans enjoy access to *food security*. According to Objective XXII, the State shall a) take appropriate steps to encourage people to grow and store adequate food, and b) establish national food reserves. At the national level, the State has not committed adequate budget allocations towards the implementation of policies, plans and regulations relevant for the realization of good nutritional health and wellbeing of vulnerable children living in children’s homes. Also, resources that enable food security, such as land and other natural resources, are under threat due to acquisition by multinational companies and lack of protection by the State. Uganda has large debts to international lending agencies, and the present research did not include a comprehensive budget analysis. It is however clear that the State of Uganda does not comply with its minimum core obligation under the CESCR [22] Article 11.2(a) to *ensure all children freedom from hunger*. According to the National Council for Children [36], the prioritization of children in budget allocations is not likely to change soon.

There were financial, human and organizational resource constraints in all institutions involved in the present study. Duty bearers reported of being dependent on external donor funding as central State funding was inadequate for State agencies and absent for civil society organizations. Most children’s home respondents perceived that there were financial resource constraints in their respective home, which may compromise the vulnerable children’s right to food and nutritional health and wellbeing, including inadequate number of staff.

Most duty bearer institutions lacked access to the necessary human resources that are required to be able to meet their obligations and duties. Such human resources are applied in the daily work with the vulnerable children, to mobilize resources, in communication strategies such as advocacy, awareness rising, empowerment, and lobbying, as well as in coordination and monitoring.

All duty bearers in the children’s homes claimed the vulnerable children have access to available mechanisms to provide feedback and exercising influence on decision-making, an essential part of the principle of the best interest of the child. However, in several of the homes these mechanisms were deemed inadequate. As regards awareness of accountability mechanisms, these were not well developed by the children’s homes.

Capacity IV involves the ability to participate in communication and to achieve a common understanding and influence decision-making and empower people to claim their rights. Our findings indicated that the communication capacity is applied in the daily work with the vulnerable children, to mobilize resources, in communication strategies such as advocacy, awareness rising, empowerment, and lobbying, as well as in coordination and monitoring activities. The findings also indicated that the legislation, policies, regulations and other relevant information agreeing with the Alternative Care Framework [13], and the human right to adequate food and related rights of the vulnerable children, were not routinely and well enough disseminated nor were they adequately sensitized towards duty bearers, the public, or relevant stakeholders. As such, mechanisms for adequate communication coordination and transparency, as well as resources and capabilities for communication within and between relevant Government institutions, civil society organizations, children’s homes, the public and stakeholders, were deemed inadequate. According to Article 24.2e of the Convention on the Rights of the Child [23], States shall ensure that all segments of society are informed and are supported in the use of basic knowledge of child health and nutrition. It was also evident that the advocacy efforts of raising the prestige of vulnerable children had not gained adequate results as evident in the continued insufficiency of Government budget allocations.

Evidence-based information on vulnerable children, the human rights of the child, and nutrition was apparently not easily available in relevant languages. However, the Ministry of Gender, Labour and Social Development had developed several tools and training manuals for caregivers, caretakers and service providers of vulnerable children, and it had developed staff guides and performance appraisals. It is important to notice that while most of the relevant Government institutions and civil society organizations were rather good at sharing information through their internet pages, most Ugandans do not have access to this information nor have the literacy skills to process such information. A broad strategy of sharing information and for capacity development of duty bearers at all levels of proximity to the child, is necessary to decrease the dependency on children’s homes and other civil society organizations, and to shift the focus over to generating livelihoods and on family and community empowerment. As regards the right to adequate food and nutrition, there is also a lack of legislation.

With regard to capacity V, having capabilities for informed rational decision-making, is key for duty bearers in the process of meeting and fulfilling their duties. Our findings indicated that the concept of the human rights based approach for good governance is not well understood among Ugandans, and that the State of Uganda has adverse governance issues such as lack of accountability, transparency, and corruption. Monitoring and evaluation efforts of vulnerable children, poverty, malnutrition, and food insecurity at State level was carried out periodically by the Uganda Bureau of Statistics, while the Ministry of Gender, Labour and Social Development, the Uganda Human Rights Commission, and the National Council for Children also performed smaller surveys in relation to vulnerable children and children’s homes. As such, all actors have access to information that makes it able to assess the problem, analyze its causes and consequences and act accordingly. However, terminology differences between different organizations create unclear statistics. This lack of clear information is a major obstacle as regards decision-making and in allocation of appropriate resources and the determination of appropriate responses.

All respondents considered child empowerment, participation and freedom of expression, and opportunities for exercising influence in decision-making, as positive. Some respondents claimed that vulnerable children merely were consulted and did not have a significant influence on the process of setting priorities for policy and public expenditure allocation. According to the National Council for Children [36], meaningful child participation is not well understood nor are their views respected. Accordingly, there were needs for budgeting geared towards changing the cultural norms and attitudes of the society as well as awareness rising of the society on meaningful child participation.

## Conclusions

We conclude that the efforts of the State of Uganda to realize its human rights obligations towards vulnerable children receiving care in children’s homes are lacking in important areas. The State of Uganda does not fulfill its minimum obligations under the CESCR to ensure all children freedom from hunger. Our findings reaffirm the need for capacity development at all levels in the Ugandan society to delimit capacity gaps in order to realize these human rights’ obligations.

## Additional files

Additional file 1 Information on relevant international treaties, general comments as well as national legislation and policy framework included in 4 tables. (PDF 389 kb)

Additional file 2 Interview guide for staff working in the children’s homes. (PDF 294 kb)

Additional file 3 Structured questionnaire for staff working in the children’s homes. (PDF 261 kb)

Additional file 4 Guide for study of equity gaps in the children’s homes. (PDF 408 kb)
