# Supplementary material for: Examination of the roles and capacities of duty bearers responsible for protecting the human rights to adequate food, nutritional health and wellbeing in Ugandan children’s homes
Source: BMC Int Health Hum Rights. 2018 Apr 17;18:17. doi: 10.1186/s12914-018-0156-4 (PMC5905179; doi:10.1186/s12914-018-0156-4)
Supplement: Supplementary file 1 — Information on relevant international treaties, general comments as well as national legislation and policy framework included in 4 tables. (PDF 389 kb) [file 12914_2018_156_MOESM1_ESM.pdf]

Additional file 1

Table 1 Selected food related provisions as established in the Universal Declaration of Human Rights, the Covenant on Economic, Social and Cultural Rights, and the Convention on the Rights of the Child. The table is developed by the authors.

United Nations 1948 Universal Declaration of Human Rights	
Art.25.1	Everyone has a right to a *standard of living adequate for the health and wellbeing of himself and of his family*, including *food*, clothing, housing and medical care and necessary social services, and the right to security in the event of unemployment, sickness, disability, widowhood, old age or other lack of livelihood in circumstances beyond his control
Art.25.2	Motherhood and *childhood are entitled to special care and assistance*. All children, whether born in or out of wedlock, shall enjoy the same social protection
United Nations 1966 Covenant on Economic, Social and Cultural Rights	
Art.9	States Parties recognize the right of everyone to *social security*, including social insurance
Art.11.1	State Parties recognize the right of everyone to an *adequate standard of living* for himself and his family, including *adequate food*, clothing and housing and the continuous improvement of living conditions. States Parties will take appropriate steps to ensure the realization of this right, recognizing to this effect the essential importance of international cooperation based on free consent
Art.11.2	States Parties, recognizing the *fundamental right of everyone to be free from hunger*, shall take, individually and through international cooperation, the measures, including specific programs, which are needed:<br><br>a) To improve methods of production, conservation and distribution of *food* by making full use of technological and scientific knowledge, by disseminating knowledge of the principles of *nutrition* and by developing or reforming agrarian systems in such a way as to achieve the most efficient development and utilization of natural resources<br><br>b) Taking into account the problems of both food-importing and food-exporting countries, to ensure an *equitable distribution of world food supplies* in relation to need
United Nations 1989 Convention on the Rights of the Child	
Art.24.2	State Parties shall pursue full implementation of [the right of the child to the enjoyment of the highest attainable standard of health] and, in particular, take appropriate measures to:<br><br>c) *Combat disease and malnutrition*, including within the framework of primary health care, through, inter alia, the application of readily available technology and through the *provision of adequate nutritious foods and clean drinking-water*, taking into consideration the dangers and risks of environmental pollution<br><br>e) Ensure that all segments of society, in particular parents and children, are informed, have access to education and are supported in the use of basic knowledge of *child health and nutrition*, the advantages of breastfeeding, hygiene and environmental sanitation and the prevention of accidents
Art.27.1	States Parties recognize the right of every child to *a standard of living adequate* for the child's physical, mental, spiritual, moral and social development
Art.27.3	States Parties, in accordance with national conditions and within their means, shall take appropriate measures to *assist* parents and others responsible for the child to implement [the right of every child to an adequate standard of living] and shall in *case of need provide* material assistance and support programs, particularly with regard to *nutrition*, clothing and housing

Table 2 Selected international treaty provisions supporting the right of the child to a standard of living adequate for development, adequate nutritious food, water, the highest attainable standard of health, and care, separated into the human rights of children and the obligations of duty bearers. The table is developed by the authors.

What are the rights holders' claims? *The right to adequate food, nutritional health and wellbeing*		
Children have the right to:		
	UDHR 25.1<br><br>UDHR 25.2	A standard of living adequate for the health and wellbeing, incl. food, clothing, housing and medical care and necessary social services, and the right to security<br><br>Motherhood and childhood are entitled to special care and assistance. All children shall enjoy the same social protection
A standard of living adequate for development	ICESCR 11.1<br>ICESCR 12.2(a)<br>ICRC 27.1	An adequate standard of living<br>Healthy development<br>A standard of living adequate for the child's physical, mental, spiritual, moral and social development
Adequate nutritious food	ICESCR 11.1<br>ICESCR 11.2<br>ICRC 24.2(c)	Adequate food<br>Be free from hunger<br>Adequate nutritious foods and clean drinking water
Water (explicit)<br>Water (implicit)	ICRC 24.2(c)<br>ICESCR 11.1<br>ICESCR 12.1	Clean drinking water<br>An adequate standard of living<br>Enjoyment of the highest attainable standard of health
The highest attainable standard of health	ICESCR 12.1<br>ICRC 24.1<br><br><br>ICRC 32.1	Enjoyment of the highest attainable standard of physical and mental health<br>Enjoyment of the highest attainable standard of health (and medical care)<br>Facilities for the treatment of illness and rehabilitation of health, and access to such health care services<br>Protection from performing any work that is likely to be harmful to the child's health or physical, mental, spiritual, moral or social development
Care	ICRC 7.1	Know and be cared for by its parents (as far as possible)
Additionally, the girl child have the right to:		
Equal access to health care services	ICEDAW 12.1; 14.2(b)	Equal access to adequate health care facilities and services incl. in rural areas and incl. those related to family planning
Equal access to information on family planning and child health and nutrition	ICEDAW 16.1(e)<br><br>ICRC 24.2(e)	Have access to information, education and means to equally enable women to decide freely and responsibly on their number and spacing of children<br>Be informed, have access to education and support in the use of basic knowledge of child health and nutrition, the advantages of breastfeeding and hygiene

What are the duty bearers' obligations?		
States Parties are obliged to:		
Realize the right to a standard of living adequate for development	ICESCR 11.1<br>ICRC 6.2<br>ICRC 27.2; 27.3	Ensure the realization of the right to an adequate standard of living<br>Ensure to the maximum extent possible the survival and development of the child<br>Assist parents and others responsible for the child to implement an adequate standard of living
Realize the right to adequate nutritious food	ICESCR 11.1<br>ICRC 24.2(c)<br>ICRC 27.3	Ensure the realization of the right to adequate food<br>Combat disease and malnutrition incl. within the framework of primary health care, and through the provision of adequate nutritious foods and clean drinking water<br>Assist parents and others responsible for the child to implement an adequate standard of living, and in case of need provide material assistance and support programs with regard to nutrition
Realize the right to the highest attainable standard of health	ICESCR 12.2 (c; d)<br>ICRC 3.3<br>ICRC 24.1<br>ICRC 24.2(b)<br>ICRC 24.3	Realize the right to prevention, treatment and control of diseases; and the right of all to medical service and attention in the event of sickness<br>Ensure provision of institutions, services and facilities which conform with appropriate standards<br>Ensure children access to health care services<br>Ensure provision of medical assistance and healthcare to all children<br>with emphasis on the development of primary healthcare<br>Abolishing traditional practices prejudicial to the health of children
Realize the right to care	ICESCR 10.1<br>ICEDAW 11.2(c)<br>ICRC 3.2<br>ICRC 3.3<br>ICRC 7.2<br>ICEDAW 16.1(d)<br>ICRC 18.1	Accord the widest possible protection and assistance to the family, particularly while it is responsible for the care and education of dependent children<br>Promote the establishment and development of childcare facilities<br>Ensure the child such care as is necessary for its wellbeing<br>Ensure that institutions, services and facilities responsible for the care of children shall conform with the standards established by competent authorities, particularly in the areas of safety, health, in the number and suitability of their staff, as well as competent supervision<br>Ensure implementation of the right of the child to know and be cared for by its parents (as far as possible) in accordance with national law<br>and relevant international instruments<br>Ensure on a basis of equality, the same rights and responsibilities as parents, irrespective of marital status, in matters relating to their children; in all cases the interests of the children shall be paramount<br>Ensure recognition of the principle that both of the parents, or legal guardians, have common primary responsibilities for the upbringing and development of the child. The best interests of the child will be their basic concern

	ICRC 18.2<br><br>ICRC 18.3	Render appropriate assistance to parents and legal guardians in the performance of their childrearing responsibilities, and to ensure the development of institutions, facilities and services for the care of children<br><br>Ensure children of working parents to benefit from childcare services and facilities for which they are eligible
Additionally, as regards the rights of the girl child, States Parties are obliged to:		
Realize the right of the girl child to equal access to health care services and nutrition	ICEDAW 12.1<br><br>ICEDAW 14.2(b)	Eliminate discrimination against women in the field of healthcare, and ensure equal access to healthcare services incl. those related to family planning<br><br>Ensure women in rural areas access adequate healthcare facilities, incl. information, counseling and services in family planning
Realize the right of the girl child to equal access to information on family planning, and child health and nutrition	ICEDAW 10(h)<br><br>ICEDAW 16.1(e)<br><br>ICRC 24.2(e)	Ensure equal access to specific educational information to help to ensure the health and wellbeing of families, incl. information and advice on family planning<br><br>Ensure women equal rights to decide freely and responsibly on the number and spacing of their children and to have access to the information, education and means to enable them to exercise these rights<br><br>Ensure that all segments of society are informed, have access to education and support in the use of basic knowledge of child health and nutrition, the advantages of breastfeeding and hygiene

Table 3 Selected provisions on the best interest of the child, in accordance with the views of the child, as established in the Convention on the Rights of the Child. The table is developed by the authors.

United Nations 1989 International Convention on the Rights of the Child	
Art.3.1	In all actions concerning children, whether undertaken by public or private social welfare institutions, courts of law, administrative authorities or legislative bodies, *the best interests of the child shall be a primary consideration*
Art.3.2	States Parties undertake to ensure the child *such protection and care as is necessary for his or her well-being*, taking into account the rights and duties of his or her parents, legal guardians, or other individuals legally responsible for him or her, and, to this end, shall take all appropriate legislative and administrative measures
Art.3.3	States Parties shall ensure that the *institutions, services and facilities responsible for the care or protection of children* shall conform with the standards established by competent authorities, particularly in the areas of safety, health, in the number and suitability of their staff, as well as competent supervision
Art. 7.2	States Parties shall ensure the implementation of [as far as possible, the *right of the child to know and be cared for by its parents]* in accordance with their national law and their obligations under the relevant international instruments…
Art.9.1	States Parties shall ensure that *a child shall not be separated from its parents against their will, except* when competent authorities subject to judicial review determine, in accordance with applicable law and procedures, that such separation is necessary for *the best interests of the child*. Such determination may be necessary in a particular case such as one involving abuse or neglect of the child by the parents, or one where the parents are living separately and a decision must be made as to the child's place of residence
Art.9.3	States Parties shall respect the right of the child who is separated from one or both parents to *maintain personal relations and direct contact with both parents on a regular basis, except* if it is *contrary to the child's best interests*
Art.18.1	States Parties shall use their best efforts to ensure recognition of the principle that both parents have common responsibilities for the upbringing and development of the child. *Parents or, as the case may be, legal guardians, have the primary responsibility for the upbringing and development of the child. The best interests of the child will be their basic concern*
Art.20.1	A child temporarily or permanently deprived of its family environment, or in whose *own best interests* cannot be allowed to remain in that environment, shall be *entitled to special protection and assistance provided by the State*
Art.21	States Parties that recognize and/or permit the system of *adoption* shall ensure that *the best interests of the child shall be the paramount consideration*
Art.12.1	States Parties shall assure to the child who is capable of forming its own views the right to express those views freely in all matters affecting the child, the *views of the child* being given due weight in accordance with the age and maturity of the child
Art.12.2	For this purpose, the child shall in particular be *provided the opportunity to be heard* in any judicial and administrative proceedings affecting the child, either directly, or through a representative or an appropriate body, in a manner consistent with the procedural rules of national law

Table 4 Selected food related provisions as found in the Ugandan Constitution, Children Act, and Penal Code Act. The table is developed by the authors.

1995 Constitution of the Republic of Uganda	
Obj. XIV	*General social and economic objectives*;<br><br>The State shall *endeavour to fulfil* the fundament rights of all Ugandans to *social justice and economic development* and shall, in particular ensure that all;<br><br>a) Development efforts are directed at *ensuring the maximum social and cultural wellbeing* of the people<br><br>b) Ugandans enjoy rights and opportunities and access to education, health services, *clean and safe water*, work, decent shelter, adequate clothing, *food security*, pension and retirement benefits
Obj. XXI	*Clean and safe water*;<br><br>The State shall take all practical measures to promote a good *water* management system at all levels
Obj. XXII	*Food security and nutrition*; The State shall;<br><br>a) Take appropriate steps to *encourage people to grow and store adequate food*<br><br>b) *Establish national food reserves*<br><br>c) *Encourage and promote proper nutrition* through mass education and other appropriate means in order to build a healthy State
Obj. XXVIII	*Foreign policy objectives*;<br><br>1) The foreign policy of Uganda shall be based on the principles of:<br><br>a) *Promotion* of the national interest of Uganda<br><br>b) *Respect* for international law and treaty obligations
Ch.2	*National Interest;* (1995 Constitution, as amended on 15 February 2006)
Art.8A	1) Uganda shall be governed based on *principles of national interest and common good* enshrined in the NODPSP<br><br>2) Parliament shall make relevant laws for purposes of giving full effect to clause 1) of this Article
Ch.4	*Rights of children;*
Art.34	1) Subject to laws enacted in their *best interests*, children shall have the right to know and *be cared for* by their parents or those entitled by law to bring them up<br><br>3) No child shall be deprived by any person of medical treatment, education or any other *social or economic benefit* by reason of religious or other beliefs<br><br>7) The law shall accord *special protection to orphans and other vulnerable children*
Ch.4	*Human rights and freedoms additional to other rights;*
Art.45	The rights, duties, declarations and guarantees relating to the fundamental and other human rights and freedoms specifically mentioned in this Chapter *shall not be regarded as excluding others not specifically mentioned*

1996 Children Act	
Art.5	*Duty to maintain a child;*
	1) It shall be the *duty of a parent, guardian or any person having custody of a child to maintain that child* and, in particular, that duty gives a child the right to: education and guidance; immunisation; *adequate diet*; clothing; shelter; and medical attention
	2) *Any person having custody of a child shall protect the child* from discrimination, violence, abuse and *neglect*
Art.7	*Harmful customary practices;*
	It shall be unlawful to subject a child to *social or customary practices* that are harmful to the child's health
Art.11	*Duty to report infringement of child's rights*;
	1) Any member of the community who has evidence that a child's rights are being infringed or that a parent, a guardian or any person having custody of a child is able to but refuses or neglects to *provide the child with adequate food*, shelter, clothing, medical care or education shall report the matter to the local government council of the area
	2) …a decision shall be made by the secretary for children's affairs in *the best interests of the child*
1950 Penal Code Act	
Ch.XV	*Desertion of children*;
Section 156	Any person who…having the lawful care or charge of a child under the age of fourteen years, and being able to maintain such child, willfully and without lawful or reasonable cause *deserts the child and leaves it without means of support*, commits a misdemeanour[1]
Ch.XV	*Neglecting to provide food, etc. for children;*
Section 157	Any person who…having the lawful care or charge of any child of tender years and unable to provide for itself, [who] *refuses or neglects,* being able to do so, *to provide sufficient food*, clothes, bedding and other necessaries for such child, so as thereby to injure the health of the child, commits a misdemeanour
Ch.XV	*Master not providing for servants or apprentices;*
Section 158	Any person who, being legally liable either as master or mistress *to provide for any apprentice or servant necessary food*, clothing or lodging, willfully and without lawful excuse refuses or neglects to provide the same…commits a misdemeanour
Ch.XIX	*Duty of head of family;*
Section 200	It is the duty of every person who, as head of a family, has charge of a child under the age of fourteen years, being a member of his or her household, to *provide the necessaries of life* for such child; and he or she shall be deemed to have caused any consequences which adversely affect the life or health of the child by reason of any omission to perform that duty, whether the child is helpless or not

[1] A misdemeanour is punishable by a moderate penalty (fine, short term imprisonment, community service). A felony is punishable by a more severe penalty.
