# Supplementary material for: Examination of the roles and capacities of duty bearers responsible for protecting the human rights to adequate food, nutritional health and wellbeing in Ugandan children’s homes
Source: BMC Int Health Hum Rights. 2018 Apr 17;18:17. doi: 10.1186/s12914-018-0156-4 (PMC5905179; doi:10.1186/s12914-018-0156-4)
Supplement: Supplementary file 2 — Interview guide for staff working in the children’s homes. (PDF 294 kb) [file 12914_2018_156_MOESM2_ESM.pdf]

ADDITIONAL FILE 2

PROTECTION OF CHILDREN`S RIGHT TO ADEQUATE FOOD, NUTRITIONAL HEALTH AND WELLBEING: A CASE STUDY OF ALTERATIVE CARE IN SELECTED APPROVED HOMES IN KAMPALA EXTRA REGION, UGANDA

Interview guide: staff working in children`s homes

Name of interviewer: ______________ Name of writer: ______________
Name of institution: ______________
Place of interview: ______________
Day: ______________ Date: ______________ Time: ______________

Personal charachteristics

Respondent name: ______________ Respondent ID: ☐
Gender: Female: ☐ Male: ☐
Position held by respondent: ______________
Highest level of completed education: Primary: ☐ Secondary: ☐ Tertiary: ☐
Education relevant for your current position: ______________
Earlier relevant positions: ______________

How long have you had your current position: ☐ years

Interview guide: role and capacity analysis

Capacity: motivation, commitment and acceptance of duty

Human rights principle: general knowledge and awareness of human rights and personal duties ("rule of law")

1.
   a. What do you consider that it involves to *support and respect* children`s human right to adequate food?

   b. What do you consider that it involves to *support and respect* children`s human right to nutritional health and wellbeing?

Probe: What *legal* responsibilities does this impose on you towards the children living here?
What *moral* responsibilities does this impose on you towards the children living here?

2. Can you mention any *international* or *regional* human rights instruments relevant for children and their right to food?

   Yes ☐ No ☐ Elaborate:

Probe: International Covenant on Economic, Social and Cultural Rights (ICESCR) and its GC12
International Convention on the Elimination of all forms of Discrimination Against Women (ICEDAW)
International Convention on the Rights of the Child (ICRC)
African Charter on the Rights and Welfare of the Child

Protocol to the African Charter on Human and Peoples Rights on the Rights of Women in Africa

3. Can you mention any *national* legislation or strategies relevant for orphans and other vulnerable children living in children`s homes, and their right to food?

Yes ☐ No ☐ Elaborate:

Probe: 1995 Constitution of the Republic of Uganda

Uganda Children`s Act

Approved Home Regulations

Uganda Nutrition Action Plan (UNAP), regarding children and adolescent girls

Alternative Care Framework

National Strategic Programme Plan of Interventions for Orphans and Other Vulnerable Children (NSPPI-2)

Draft Uganda Food and Nutrition Policy and Strategy (UFNP/UFNS)

<u>If yes:</u> Can you describe any relevant provisions?

---

4. Can you mention which *Ministry* is responsible for vulnerable children, including children in institutional care?

Yes ☐ No ☐

---

5. In this project, capacity entails motivation, authority, resources (economic, human, and organizational), communication, and decision making.

How can you use these capacities in meeting your duties of realizing the rights to food of the children living here?

---

6. What *type of food* do you consider as good for children`s health and wellbeing?

Probe: Why?

How will *adequate* food affect children?

How will *inadequate* food affect children?

Traditionally, how was this perceived in your *own community*? Do you think this has *changed*?

---

7. Do you consider the *food, health and care provision* in the children`s home as adequate?

Yes ☐ No ☐ Don't know ☐ Elaborate:

Probe: In which areas could it be *improved*?

Human rights principle: internalization of basic human rights standards, values and principles, and acceptance of personal obligations ("accountability, respect for the human rights and the rule of law, dignity")

---

8. What do children *need* from their caretakers?

Probe: Food, health, care, protection, respect, support, love

---

9.

a. What does *child vulnerability* entails?

Probe: *Which* children in Uganda are vulnerable?

b. What does *orphanhood* entails?

Probe: Can children with *one or two living parent* be orphans?

Yes ☐ No ☐ Don't know ☐ Why:

10. Is the Ugandan *girl child more vulnerable* than the boy child?

Yes ☐ No ☐ Don’t know ☐ Why:

---

11. Have you heard about the “*window of opportunity*” and the “*life course approach*” in relation to eradication of hunger and child malnutrition?

Yes ☐ No ☐ Yes, but don’t remember what it entails ☐ Elaborate:

---

12. Does the home have a *distinctive focus* on the special nutritional, health and care needs of adolescent girls?

Yes ☐ No ☐ Don’t know ☐ Elaborate:

Probe: Is the *food provided* to adolescent girls adequate to support growth, development and future childbearing?

Yes ☐ No ☐ Don’t know ☐ Elaborate:

---

13. What is your impression of what the food and the mealtimes *mean* for the children?

Probe: *What contributes* to a positive experience for the children during mealtimes?

Could anything be *different* in the organization of the meals and mealtimes?

---

14. *What do you do* if you discover that a child is malnourished or are not eating or drinking enough?

Probe: What could be *different in the routines* for identification of child malnutrition?

What could be *different in the organization* of the health and care services provided to the children?

---

15. Non-discrimination, access to information, participation, accountability, and sustainability are fundamental human rights principles.

Can you mention how you use these principles in your daily work with the children?

Human rights principle: non-discrimination, equity, equality and human dignity

---

16. Do you think discrimination may *affect the access* to food, water and healthcare of the children living here?

Yes ☐ No ☐ Don’t know ☐ How:

Probe: The girl child

---

17. Have you heard about *affirmative action* or *positive discrimination* in order to address existing discrimination?

Yes ☐ No ☐ Yes, but don’t remember what it entails ☐

Probe: How can affirmative action be *actively used* in your work, in order to address existing discrimination in relation to the right to adequate food?

Youngest children

Adolescent girls

Human rights principle on children`s rights; society`s internalization of basic human rights standards, values, principles and duties (“accountability, responsibility of leaders and individuals, respect for the human rights and the rule of law,

---

dignity, sustainability")

18. We are now interested to know your perceptions regarding the *role of the child within the family*:

a. Can you describe the *society`s attitudes* regarding intolerable behaviour towards punishing children as a part of child raising?

Probe: What is *intolerable* behaviour?

What is *tolerable* behaviour?

Is it accepted in the society to *deny a child access to food for a short period* if the child has been naughty?

Yes ☐ No ☐ Don't know ☐ Elaborate:

b. How is the *society`s attitudes* regarding the role of the child within the household today?

Probe: What is the *child`s role* within the household?

What *expectations* lie on the child?

Is the child normally given *special priority* of food provision within the household?

Yes ☐ No ☐ Don't know ☐ Elaborate:

Probe: What does household *food and nutrition security* for children entails?

How may the society`s attitudes affect children`s level of household *food and nutrition security*?

---

19. Compared with the traditional social security mechanisms through the extended families, how is the *society`s perceptions* regarding the *level of food and nutrition security* for the children living in children`s homes, and regarding *practices* that may affect their wellbeing?

Probe: Do the society consider the children living in children`s homes as *more or less* food and nutrition secure than other vulnerable children?

Is there any *practices* you consider may affect the wellbeing of children living in children`s homes?

---

20. We are now interested to know your perceptions regarding *human rights in the Ugandan context*:

a. Do you think human rights are *applicable* in the Ugandan context?

Yes ☐ No ☐ Don't know ☐ Why:

b. Do you think human right to adequate food of orphans and other vulnerable children living in children`s homes are *applicable* in the Ugandan context?

Yes ☐ No ☐ Don't know ☐ Why:

c. How do you consider the *applicability* of the international compared with the African regional human rights instruments in the Ugandan context?

Probe: With emphasis on the right to adequate food of children living in children`s homes?

---

21. Are there any traditional beliefs, customs, norms, rituals, or hierarchies in the society, which may *interfere* with optimal food practices?

Yes ☐ No ☐ Don't know ☐ Elaborate:

Probe: How could these potentially *affect children* living in children`s homes?

How could these potentially *affect the girl child*?

22. We are now interested to know your perceptions regarding *food taboos*:

a. Do you think food taboos are still *existing* and *affecting* some adolescent girls in the country today?

Yes ☐ No ☐ Don't know ☐ Elaborate:

Probe: What is your *perception of food taboos* in relation to the nutritional health of adolescent girls?

b. Do you think food taboos today may be a potential *limiting factor* for the nutritional health of adolescent girl's living in children`s homes throughout the country?

Yes ☐ No ☐ Don't know ☐ How:

c. What do you think of cultural and/or religious institutions in the community *wishing to contribute* in the upbringing of children living in children`s homes?

Probe: Do you think cultural, traditional and religious norms and values affecting the nutritional health of children may be used to *serve ulterior motives* by civil society organizations?

Yes ☐ No ☐ Don't know ☐ How:

Human rights principle of children`s rights; society`s internalization of basic human rights standards, values, principles and duties ("accountability, responsibility of leaders and individuals, respect for the human rights and the rule of law, dignity, sustainability")

---

23. We are now interested to know your perceptions regarding the *extended family*:

a. Can you mention what practices that already exist in the society to protect, care, and support orphans and other vulnerable children?

b. How are the traditional roles of the extended family *changing*?

c. What expectations *still* lie on the extended family?

d. What can be done to *support the extended family* in its traditional roles towards protecting and care for orphans and other vulnerable children?

e. How do you think the *society feel* about orphaned and other vulnerable children?

Probe: Positive and negative feelings

24. We are now interested to know your perceptions regarding *children`s homes*:

a. What do you consider as the *benefits* of children`s homes?

b. What do you consider as the *problems* of children`s homes?

c. How do you think the *society feel* about children`s homes, and the *need* for these institutions?

Probe: Positive and negative feelings

d. How would you *explain the attitudes* of parents and extended families contributing to the practice of sending both *orphaned and non-orphaned* children to live in children`s homes?

e. Do you think child vulnerability and orphanhood is being *socially constructed* through the creation of children`s homes?

Yes ☐ No ☐ Don't know ☐ How:

Capacity: legal, political, social and cultural authority to make decisions and to take action

Human rights principle: the opportunities, restrictions and/or limitations for self-assertion (exercising influence) ("accountability, responsibility of leaders and individuals, respect for the rule of law")

25. Who do you consider are *responsible* for safeguarding the right to adequate food of the children living here?

Probe: Legally: Government level
Morally: Civil society level
Children`s home level

26. Can you describe *your areas of authority and responsibilities* in the children`s home?

Probe: Are you given *adequate authority* to implement and enforce relevant strategies?

What *constrains* do you meet in fulfilling your duties towards the children`s right to food and nutritional health?

How can structures of *authority hierarchy* compromise (or benefit) the children`s rights to adequate food and nutritional health?

How do you work with these challenges?

27. How do you go about it if you wish to change routines or procedures?

Probe: What *challenges* can you meet?

28. How are your supervisors *encouraging* you to participate more effectively to harness your potential and to fulfill your duties towards the children?

Capacity: availability, access and control over relevant economic, human and organizational resources to enable decision making and action

Management of economic resources

Human rights principle: economic resources of the children`s home (available and accessible to, and control by, relevant staff) ("transparency, accountability, responsibility of leaders and individuals, respect for the rule of law, sustainability")

29. Does the State have *obligations to realize* the right to adequate food for children living in children`s homes?

Yes ☐ No ☐ Don't know ☐ Why:

Probe: If yes: *How* should the State exercise these obligations?

30. Does the State have *obligations to provide* food and supplementation for malnourished children living in children`s homes?

Yes ☐ No ☐ Don't know ☐ Why:

Probe: If yes: *How* should the State implement these obligations?

---

31. Does non-State actors have *duties to provide* food for children living in children`s homes?

Yes ☐ No ☐ Don't know ☐ Why:

Probe: If yes: *How* should non-State actors exercise these duties?

If yes: *How* should the State enforce these duties of the non-State actors?

---

32. Are there any *financial resource constraints*, which may compromise the children`s right to food and nutritional health?

Yes ☐ No ☐ Don't know ☐ Elaborate:

Probe: If yes: How is basic need provisions *prioritized*?

If yes: How are the needs of the *most vulnerable* children safeguarded?

In case of lack of economic resources, how will the children`s home go about it to *generate more funding*?

Management of human resources

Human rights principle: human resources of the children`s home (available and accessible to, and control by, relevant staff) ("transparency, accountability, responsibility of leaders and individuals, respect for the rule of law, sustainability")

---

33. How are you working to *oversee* the implementation and monitoring of strategies for realizing the children`s right to adequate food and nutritional health?

---

34. *Where* have you learned what you know about human rights and childrens need for food, health and care?

Probe: How has the children`s home or the State *contributed* to increase your capacity, performance and interest in the field of human rights and children`s right to adequate food and nutritional health?

Management of organizational resources

Human rights principle: organizational resources of the children`s home (available and accessible to, and control by, relevant staff) ("transparency, accountability, responsibility of leaders and individuals, respect for the rule of law, sustainability")

---

35. Does the children`s home regularly monitor and evaluating its *own performance* in accordance with national policy criteria?

Yes ☐ No ☐ Don't know ☐ Elaborate:

Probe: If yes: Who is responsible?

How often/when was the last time?

Which gaps were identified?

What actions have been undertaken to improve performance?

---

36. Are you aware of any mechanisms that effectively can hold duty bearers *accountable* for inadequate or non-delivery of services towards children?

Yes ☐ No ☐ Elaborate:

---

37. Has the children`s home established mechanisms for the *children to provide feedback* and exercising influence on decision making?

Yes ☐ No ☐ Don't know ☐ Elaborate:

Probe: <u>If yes:</u> What are the number and sort of feedback received?

<u>If yes:</u> Has any of the feedback involved lack of adequate provision of food, health, care and protection?

Capacity: resources and capabilities for effective communication

Human rights principle: capabilities of the children`s home to communicate effectively ("participation, involvement")

38.

a. Why do you think there *exists* so many children`s homes in Uganda?

b. Why do you think so many children`s homes are *not ensuring compliance* with national legislation?

c. Why do you think there is *engagement limitations* between children`s homes and the local Government?

39. What do you think about family and extended family reunification of orphans and other vulnerable children living in children`s homes?

Probe: Positive/negative

How can the children`s home work to increase the practice?

40. What do you think about national adoption of orphans and other vulnerable children living in children`s homes?

Probe: Positive/negative

How can the children`s home work to increase the practice?

41. What do you think about international adoption of orphans and other vulnerable children living in children`s homes?

Probe: Positive/negative

How can the children`s home work to decrease the practice?

Human rights principle: access to information (seek, receive and impart) concerning the human rights of the child ("child empowerment")

42. Is it desirable to *promote* children`s awareness of their rights to adequate food and related rights, so they hence can access redress if their rights are violated?

Yes ☐ No ☐ Don't know ☐ Why:

Probe: <u>If yes:</u> How are *you* working to increase the children`s awareness of their human rights?

What *mechanisms* does this children`s home have in place for the children to be able to claim their rights?

Capacity: capabilities for rational decision making and leaning

Human rights principle: capabilities of children`s homes for informed and rational decision making and learning from experience ("accountability, responsibility of leaders and individuals, sustainability")

43. Is the children`s home following the *human rights approach to good governance* to obtain good nutritional health and wellbeing of the children living here?

Yes ☐ No ☐ Don't know ☐ How:

Human rights principle: child freedom of expression and opportunities for exercising influence, through inclusion, involvement and participation in decision making processes concerning the human rights of the child ("child empowerment")

44. What mechanisms are in place for *including the children* and the concerned stakeholders in decision making relevant for their lives?

Probe: Are these mechanisms *adequate*?

Yes ☐ No ☐ Don't know ☐ Elaborate:

Finishing questions:

45. What do you consider as *positive and functional* about Uganda`s system of safeguarding children and their right to good nutritional health?

46. Is there anything you would *like to add* that we have not discussed, and that you consider could be valuable for this study?

47. Do you have any *suggestions* regarding how this institution could *change procedures* to better comply with the principle of the best interest of the child and their right to adequate food and nutritional health?

Thank you very much for the interview!
