# Supplementary material for: Examination of the roles and capacities of duty bearers responsible for protecting the human rights to adequate food, nutritional health and wellbeing in Ugandan children’s homes
Source: BMC Int Health Hum Rights. 2018 Apr 17;18:17. doi: 10.1186/s12914-018-0156-4 (PMC5905179; doi:10.1186/s12914-018-0156-4)
Supplement: Supplementary file 3 — Structured questionnaire for staff working in the children’s homes. (PDF 261 kb) [file 12914_2018_156_MOESM3_ESM.pdf]

ADDITIONAL FILE 3

PROTECTION OF CHILDREN`S RIGHT TO ADEQUATE FOOD, NUTRITIONAL HEALTH AND WELLBEING: A CASE STUDY OF ALTERATIVE CARE IN SELECTED APPROVED HOMES IN KAMPALA EXTRA REGION, UGANDA

Structured questionnaire: staff working in children`s homes

Name of institution: ______________________

Day: ______________ Date: ______________ Time: ______________

Personal charachteristics

Respondent name: ______________________ Respondent ID: ☐

Gender: Female: ☐ Male: ☐

Position held by respondent: ______________________

Highest level of completed education: Primary: ☐ Secondary: ☐ Tertiary: ☐

Education relevant for your current position: ______________________

Earlier relevant positions: ______________________

How long have you had your current position: ☐ years

Self-administrated structured questionnaire: role and capacity analysis

Capacity: motivation, commitment and acceptance of duty

Human rights principle: general knowledge and awareness of human rights and personal duties (“rule of law”)

	Yes	No	Don’t know
1. Are you satisfied with the following in the children’s home:			
a. The adequacy of food provided to the children?			
b. The adequacy of healthcare provided to the children?			
c. The adequacy of care and protection provided to the children?			

Comments:

Human rights principle: internalization of basic human rights standards, values and principles, and acceptance of personal obligations (“accountability, respect for the human rights and the rule of law, dignity”)

Human rights principle: non-discrimination, equity, equality and human dignity

	Yes	No	Don’t know
2. Is there any difference between the following in the children’s home:			
a. The access and availability of food for the girl and the boy child?			
b. The access to healthcare and nutritional healthcare for the girl and the boy child?			
c. The level/rate of malnutrition among the girl and the boy child?			

Comments:

	Yes	No	Don’t know
3. Does the children’s home have a distinctive focus on the special needs (nutritional, health, care) of the adolescent girls?			
a. Do the children receive any supplementation with micro- and/or macronutrients? If yes:			
Is increased energy part of the supplementation?			
Is increased vitamin A part of the supplementation?			

Is increased iron part of the supplementation?			
b. Are the adolescent girls receiving routinely health checks?			
With special focus on underweight?			
With special focus on vitamin A deficiency?			
With special focus on anemia?			
c. Do you consider the adolescent girls to be adequately cared for?			

Human rights principle on children`s rights; society`s internalization of basic human rights standards, values, principles and duties ("accountability, responsibility of leaders and individuals, respect for the human rights and the rule of law, dignity, sustainability")

Human rights principle of children`s rights; society`s internalization of basic human rights standards, values, principles and duties ("accountability, responsibility of leaders and individuals, respect for the human rights and the rule of law, dignity, sustainability")

Capacity: legal, political, social and cultural authority to make decisions and to take action

Human rights principle: the opportunities, restrictions and/or limitations for self-assertion (exercising influence) ("accountability, responsibility of leaders and individuals, respect for the rule of law")

Capacity: availability, access and control over relevant economic, human and organizational resources to enable decision making and action

Management of economic resources

Human rights principle: economic resources of the children`s home (available and accessible to, and control by, relevant staff) ("transparency, accountability, responsibility of leaders and individuals, respect for the rule of law, sustainability")

4. Are you satisfied with the sufficiency of financial resources regarding the following in the children's home:	Yes	No	Don't know
The budget available for realizing the right to adequate food and nutritional health and wellbeing of the children?			
The economic resources available to ensure adequate employment of relevant staff?			
The economic resources available to ensure proper training of all staff in human rights, nutrition, health and care, and management of the orphans and other vulnerable children?			

<u>Comments:</u>

5. Are you satisfied with the prioritizing of financial resources regarding the following in the children's home:	Yes	No	Don't know
a. The provision of food, health and care for good nutrition to the children?			
b. Do you consider the relative share of funds allocated to this area as reasonable?			

<u>Comments:</u>

6. Are you satisfied with the adequacy of the following in the children's home:	Yes	No	Don't know
a. The extent of transparency in the use of resources?			
b. The extent of accountability in the use of resources?			

<u>Comments:</u>

7. In the past 4 weeks, did it happen that the children in the children's home:	Yes	No	Don't know
a. Were not able to eat the kinds of food you would have preferred for them to eat, because of lack of resources?			
b. Had to eat a limited variety of foods because of lack of resources?			
c. Had to eat a smaller meal than they needed because there was not enough food?			
d. Had to eat fewer meals in a day because there was not enough food?			
e. Went to sleep at night hungry because there was not enough food?			

	Question	Yes	No	Don't know
f.	Went a whole day and night without eating anything at all because there was not enough food?			
g.	In the past 4 weeks, did it happen that there was no food to eat of any kind in the children's home, because of lack of resources?			
h.	Do you consider the last 4 weeks as representative for the previous 12 months of availability of nutritious foods, for the children living in the children's home?			

Comments:

8.		Yes	No	Don't know
a.	Is it desirable that the State builds and operates children`s homes?			
b.	Is it desirable that the State increase the control of children`s homes, and limit their operation?			
c.	Does the State have legal obligations to provide food and supplementation for malnourished children living in children`s homes?			
d.	Does the State have moral obligations to provide food and supplementation for malnourished children living in children`s homes?			
e.	Does non-State actors have duties to provide food for children living in children`s homes?			

Comments:

Management of human resources

Human rights principle: human resources of the children`s home (available and accessible to, and control by, relevant staff) ("transparency, accountability, responsibility of leaders and individuals, respect for the rule of law, sustainability")

9.	Are you satisfied with the adequacy of the following in the children's home:	Yes	No	Don't know
a.	The skills, experience, devotion, and available time of relevant staff?			
b.	The children`s home to facilitate an enabling environment, with satisfactory numbers of accountable and empowered staff respecting the human rights and the rule of law and the human dignity of children?			
c.	Relevant staffs' awareness of their own human rights, i.e. as rights holders and citizens, and as employees in the children`s home?			
d.	The level of knowledge and adherence to the Alternative Care Framework, the Approved Home Regulations, the National Strategic Programme Plan of Interventions for Orphans and Other Vulnerable Children (NSPPI-2) and the Uganda Nutrition Action Plan (UNAP) among staff?			

Comments:

10.	Are there any form of human resource constraints, which may compromise the children`s right to food and nutritional health?	Yes	No	Don't know

Comments:

Management of organizational resources

Human rights principle: organizational resources of the children`s home (available and accessible to, and control by, relevant staff) ("transparency, accountability, responsibility of leaders and individuals, respect for the rule of law, sustainability")

11. How often are capacity building activities undertaken in the children`s home?

Monthly: ☐ Quarterly: ☐ Biannual: ☐ Annual: Never: ☐ Don't know: ☐

Comments:

12.	Have the following issues been on the agenda, and been adequately covered:	Yes	No	Don't know
a.	Human rights of children:			

b.	Human rights standards and principles:			
c.	Children`s human right to adequate food:			
d.	Children`s human rights to health:			
e.	Children`s human rights to care:			
f.	Food security:			
g.	Nutrition security:			
h.	Nutrition:			
i.	Child malnutrition:			
j.	Nutrition and child malnutrition:			
k.	The girl child and the "window of opportunity:			
l.	Child vulnerability:			
m.	Child protection:			
n.	Non-discrimination:			
o.	The Alternative Care Framework:			
p.	Institutional care and the effects on the child:			
q.	Family reunification:			
r.	Foster care, national and international adoption:			
s.	Democratic governance and corruption:			

Comments:

		Yes	No	Don't know
13.	Do you think the State is adequately monitoring the efforts of the country`s children`s homes in meeting its duties towards the children living there?			

Comments:

Capacity: resources and capabilities for effective communication

Human rights principle: capabilities of the children`s home to communicate effectively ("participation, involvement")

		Yes	No	Don't know
14.	Do you think children`s homes are sustainable and effectual ways of raising orphans and other vulnerable children, and ensuring their right to food and nutritional health?			
15.	Are you aware that the State considers:			
	Children`s homes as unsustainable and ineffectual, and are working to limit their operation?			
	International donor funding of children`s homes as contrary to national legislation, and as undermining national development efforts?			
	Institutionalization of children to increase destitution of families, affecting orphans and other vulnerable children and their human rights?			
	Institutionalization as not in the best interests of the child, as it is damaging to their social and mental development, is undermining the rights of children to be cared for by their families, and is creating child vulnerability?			
	Do you think the State is correct in these issues?			

Comments:

		Yes	No	Don't know
16.	This children`s home is Approved by the Government.			
	Are you aware of this children`s homes response towards the Governments appeal to join and align with the Alternative Care Framework in a National coordinated response to reunite all children with their families?			

Comments:

Human rights principle: access to information (seek, receive and impart) concerning the human rights of the child ("child

empowerment")

17. Do State actors regularly interact with the children living here? / Does all staff regularly interact with the children living here? / Have State actors explored if the children living here are aware of their right to adequate food to obtain good nutritional health and wellbeing?

17.	Yes	No	Don't know
Do State actors regularly interact with the children living here?			
Does all staff regularly interact with the children living here?			
Have State actors explored if the children living here are aware of their right to adequate food to obtain good nutritional health and wellbeing?			

Comments:

18. Do you perceive the following as positive for children:	Yes	No	Don't know
a. Child empowerment?			
b. Child empowerment, through access to information relevant for their rights to obtain good nutritional health and wellbeing?			
c. Child empowerment, through capacity and awareness-raising in areas relevant for their rights to obtain good nutritional health and wellbeing?			
d. Child empowerment, through the relevant channels to access redress if their rights are abused?			
e. Child empowerment, through the encouragement of participation, freedom of expression, and opportunities for exercising influence on decision making?			

Comments:

19. Do you consider the media coverage and communication in matters regarding children`s homes and orphans and other vulnerable children, in relation to their right to adequate food and nutritional health and wellbeing as:	Yes	No	Don't know
a. Adequate?			
b. Truthful?			

Comments:

Capacity: capabilities for rational decision making and leaning

Human rights principle: capabilities of children`s homes for informed and rational decision making and learning from experience ("accountability, responsibility of leaders and individuals, sustainability")

Human rights principle: child freedom of expression and opportunities for exercising influence, through inclusion, involvement and participation in decision making processes concerning the human rights of the child ("child empowerment")

Thank you very much!
