# Supplementary material for: Examination of the roles and capacities of duty bearers responsible for protecting the human rights to adequate food, nutritional health and wellbeing in Ugandan children’s homes
Source: BMC Int Health Hum Rights. 2018 Apr 17;18:17. doi: 10.1186/s12914-018-0156-4 (PMC5905179; doi:10.1186/s12914-018-0156-4)
Supplement: Supplementary file 4 — Guide for study of equity gaps in the children’s homes. (PDF 408 kb) [file 12914_2018_156_MOESM4_ESM.pdf]

ADDITIONAL FILE 4

Guide: Equity gap study in children`s homes

Day and date: ____________________
Name of institution: ____________________
Location: ____________________

Baseline data – foundation/operation of the children`s home: inquiries to the management

1. Foundation body: ____________________
   - Religious foundation: ☐
   - Non-religious foundation: ☐
2. Year of opening: ____________________
3. Legal status - date of registration of approval: ____________________
4. Institutional policy: *(ASK FOR COPY)*
   - Vision: ____________________
   - Mission: ____________________
   - Goals and objectives: ____________________
5. Associated institutions: *(ASK FOR FULL NAME, ADDRESS, PHONE, EMAIL)*
   - NGO/CSO/CBOs: ____________________
   - Health centers: ____________________
   - Schools: ____________________
   - Adoption agencies: ____________________
   - Churches: ____________________
   - Others: ____________________
6. Sources of funding: ____________________
   Sources of donations: ____________________
7. Average number of children: ____________________
   - Age range: ____________________
   - Boy/girl ratio: ____________________
8. Directors: ____________________
   - Responsibilities: ____________________
   - How they work: ____________________

   Management Committee: *(ASK FOR COPY OF POLICY DOCUMENTS AND PROFILES)*
   - Who are members: ____________________
   - Responsibilities: ____________________
   - How they work: ____________________
9. Managers:
   - Authority: ____________________
   - Responsibilities: ____________________
   - How they work: ____________________
10. Caretakers:
    - Authority: ____________________

Responsibilities: ______________________

Total number employed: ______________________

Number present every day: ______________________

Males: ______________________

Females: ______________________

Number present every night: ______________________

Males: ______________________

Females: ______________________

Caretaker/child-ratio: ______________________

Number of volunteers: ______________________

Number of educated/trained caretakers present every day: ______________________

Qualifications required: *(SPECIFY)*

Education: ______________________

References: ______________________

Previous experience: ______________________

Specific skills: ______________________

Others: ______________________

Relevant training provided: *(SPECIFY)* ______________________

____

11. Child admission policy: *(ASK FOR COPY)*

Main reasons for child admission: ______________________

____

Description of the process/assessment of child admission, including the procedure of court orders and of health examination and vaccination: *(ASK TO SEE A SAMPLE)*

____

____

____

____

12. Services provided (meals, immunization, counseling, schooling, etc.) ______________________

____

____

Annual food menu card: *(ASK FOR COPY)*	Yes		No	
Annual food budget: *(ASK FOR COPY)*	Yes		No	
Records/documentation/file for each child: *(ASK TO SEE A SAMPLE)*	Yes		No	

13. Aim for the children in care:

Family reunification programs (parents or extended family):	Yes		No	
Support families in their homes:	Yes		No	
Foster homes programs:	Yes		No	
National adoption programs:	Yes		No	
International adoption programs:	Yes		No	
Others: *(SPECIFY)*	Yes		No	

____

____

14. Follow-up routines when the child is leaving the home: *(DESCRIBE)* ______________________

____

____

15. Other relevant information: ____________________

Equity gap study of the realization of the five human rights criteria of just availability, accessibility, acceptability, affordability and quality of the children`s rights to adequate food, water, sanitation, health, care and protection in the children`s home (observations and inquiries to relevant staff)

Food and food sources (CHEF/CATERER)

Availability;

16. Does the children`s home have:	Yes	No	Comments:
Secure food sources:			
Steady food supply:			
Sufficient quantity of food:			
Sufficient quality of food:			

17. Are there sometimes any disruption of:	Yes	No	*(DAYS/WEEKS)*
Secure food sources:			
Steady food supply:			
Sufficient quantity of food:			
Sufficient quality of food:			

18. Who are financing or providing the food:	Yes	No	*(SPECIFY)*
Ugandan Government:			
Donations/aid:			
NGOs:			
Local markets:			
The children`s home:			
Others:			

Accessibility;

19. Does the budget provide for sustainable economic access at all times to sufficient adequate food for all:	Yes	No	Comments:
Without threatening or compromising the attainment of other basic needs:			
Are there any special food programmes:			
Are there any food gardens to supplement the food requirements? *(IF YES: OBSERVATION)*			

20. Do all children have access at all times to sufficient quantity and quality of foods?	Yes	No	Comments: *(COPY OF MEAL SCHEDULES)*
Are there regular meals throughout the day:			
Can children choose from a variety of foods in accordance with their likes and dislikes:			
Can children choose to eat outside of the regular meals:			
Do children have access to food between meals and at night:			
Is there possibility for several servings during one meal:			
Are fruits and vegetables served every day:			

21. Are foods distributed equally between:	Yes	No	Comments:

	Yes	No	Comments:
Girls and boys:			
Different age groups:			
22. Are girls and young children given special attention and priority consideration in terms of diet:			
Are supplementation with micro- and/or macronutrients provided for:			
If yes: Are supplementation with micro- and/or macronutrients received:			
By all children:			
Only by malnourished children:			
Only by adolescent girls:			
Containing energy:			
Containing vitamin A:			
Containing iron:			
Containing vitamin B12:			
23. Are children involved in aspects of procurement, preparing and decision making regarding food planning and interval of mealtimes, etc.:			

Who prepare, cook and serve the food? ______________________________

____

Quality;

	Yes	No	Comments:
24. Do the children`s home have a problem of food related diseases (diarrhea, food poisoning):			

What protective measures are taken to prevent contamination of food through bad environmental hygiene or inappropriate handling?

____

What preservation and storage facilities do the children`s home have? (*OBSERVATION*)

____

Acceptability;

	Yes	No	Comments:
25. Do options exist for residents with different religious or cultural backgrounds who need customized food:			
26. Are food and mealtimes organized in culturally appropriate ways:			(*OBSERVATION*)
27. Are food and mealtimes age appropriate:			(*OBSERVATION*)
28. Are the food provided culturally appropriate:			(*OBSERVATION*)
Colour:			
Odour:			
Taste:			
29. Appropriate physical designs of kitchen area:			(*OBSERVATION*)
Appropriate hygiene conditions in the kitchen area:			
Appropriate physical designs of eating area:			
Appropriate hygiene conditions in the eating area:			

30. How are mealtimes organized to contribute to a positive experience for the children?

____

Affordability;

31. What percentage of the total budget is reserved for food? ____________

Conclusion;

32. The children`s home has sufficient adequate food and food services:

	Yes	No	Comments:
The children`s home has sufficient adequate food and food services:			

Water and water sources (CHEF/CATERER)

Availability;

33. Does the children`s home have:

	Yes	No	Comments: (*OBSERVATION*)
Secure water sources:			
Functional water sources:			
Steady water supply:			
Sufficient quantity of water:			
Sufficient quality of water:			

34. Approximately how many liters of water are available per child per day (personal/domestic use)? ____________

35. Are there sometimes any disruption of:

	Yes	No	Comments: *(HOURS/DAYS/WEEKS)*
Secure water sources:			
Steady water supply:			
Sufficient quantity of water:			
Sufficient quality of water:			
Any alternative source of water:			

If yes: where/distance? ____________

Accessibility;

	Yes	No	Comments:
36. Does the budget provide for sustainable economic access at all times to sufficient adequate water for all:			
Without threatening or compromising the attainment of other basic needs:			
37. Do all children have access at all times to sufficient quantity and quality of drinking water:			
Do children have access to water between meals and at night:			

38. Are water distributed equally between:

	Yes	No	
Girls and boys:			
Different age groups:			
39. Are children involved in procurement of water:			(*OBSERVATION*)
Water collection time don’t exceed 30 minutes:			
Distance between the children`s home and the water sources is within 1.000 metres:			
Physical security is not threatening access to the water sources:			

Quality;

	Yes	No	Comments:
40. Do the children`s home have problems of water related diseases (diarrhea):			
41. Do the children`s home take any protective measures			

to ensure the drinking water is clean and safe: ☐ ☐ ________

If yes: What protective measures are taken? ________

____

Acceptability;

42. Are the water provided culturally appropriate:

	Yes	No	Comments: (*OBSERVATION*)
Colour:			
Odour:			
Taste:			

43. Water facilities accommodates common hygiene practices:

	Yes	No	
Water facilities accommodates common hygiene practices:			
Water facilities has appropriate physical designs:			
Water facilities are properly maintained and serviced:			

Affordability;

44. What percentage of the total budget is reserved for water? ________

Conclusion;

45. The children`s home has sufficient adequate water services:

Yes	No	Comments:

____

Sanitation and hygiene (HEALTH PERSONNEL/CARETAKER)

Availability;

46. Does the children`s home provide a:

	Yes	No	Comments: (*OBSERVATION*)
Hygienic/clean indoor environment:			
Safe/hygienic outdoor environment:			
Sufficient number of sanitation facilities (toilet/washroom area):			

What is the number of people using the sanitation facility (toilet and washroom area)? ________

Accessibility;

47. The children`s home provide adequate sanitation facilities (toilet and washroom area) that are:

	Yes	No	Comments: (*OBSERVATION*)
Situated within a distance not exceeding 30 minutes of walking back and forth:			
Situated within a distance of 1.000 metres from the children`s home:			
Sited in such a way as to minimize physical security threats to the users day and night:			
Safe to use for children:			
Child-sized holes available:			
Are small children accompanied to the toilet:			
Any alternative sanitation facilities available during maintenance periods:			

If yes: where/distance? ________

Quality;

48. Toilet area is hygienically and technically safe to use, and effectively prevent human, animal and insect contact with human excreta:

	Yes	No	Comments: (*OBSERVATION*)
Toilet area is hygienically and technically safe to use, and effectively prevent human, animal and insect contact with human excreta:			
Washroom area is hygienically/technically safe to use			

	Yes	No	Comments
Toilets/washroom areas cleaned with soap/water:			
Clean water and hygiene articles (soap, washtub) are accessible for hand washing, bathing, etc.			

49. General hygiene in the children`s home:	Yes	No	Comments: (*OBSERVATION*)
Visible bugs/rodents:			
Visible trash:			
Visible animal feces:			
Floors swept and cleaned with soap and water:			
Dishes/utensils washed with soap and hot water:			

50. Do the children`s home have problems of poor hygiene related diseases (diarrhea):	Yes	No	

Acceptability;

51. Sanitation facilities (toilet and washroom area):	Yes	No	Comments: (*OBSERVATION*)
Accommodates common hygiene practices:			
Culturally/socially acceptable physical design:			
Are properly maintained and serviced:			
52. Sanitation facilities (toilet and washroom area) are sensitive to gender/lifecycle/privacy requirements:			
Separate facilities for women/men, and girls/boys:			
Adolescent girls have private space in the toilet and washroom area:			
Allow for the disposal of menstrual hygiene materials:			
Are sanitation towels provided for?			
Are other menstrual hygiene materials provided for?			
<u>If yes:</u> Is there space and time that allows for convenient washing and drying of menstrual hygiene materials?			
Do adolescent girls receive assistance and support in menstrual hygiene management?			

Affordability;

53. What percentage of the total budget is reserved for sanitation and hygiene? ______________

Does access to sanitation and hygiene services compromise the ability to pay other essential necessities such as food, housing and healthcare?	Yes	No	Comment:

Conclusion;

54. The children`s home has sufficient adequate hygiene and sanitation services:	Yes	No	Comments:

____

____

Protection and care, including healthcare services (HEALTH PERSONNEL/CARETAKER)

Availability;

	Yes	No	Comments: (*OBSERVATION*)
55. The children`s home is protected from intrusion/vandalism (fences/guards):			
56. Is the family encouraged to pay regular visits, and to take visits from the child?			
57. Does the children`s home have employed full time **primary medical personnel**?			

If no: How is primary healthcare provided?

First aid kit and medications are available, and safely secured:

Budget provides for sustainable economic operations and practice at all times of medical personnel, including necessary equipment:

Without compromising the attainment of other basic needs:

Accessibility;

58. Caretakers interact with the children (smiling, touching, talk, listen, etc.): Yes No Comments: (*OBSERVATION*)

59. What communication and information systems do you have, and who have access?

60. How often do the children get regular **medical check-ups and referrals**?

Are routinely health checks received consistently by both girls and boys? Yes No Comments:

Quality;

61. The children`s home provides for: Yes No Comments: (*OBSERVATION*)

Adequate shelter and ventilation:

The possibility for play outdoors:

Adequate supervision of all children:

A visually stimulating and encourages learning environment:

Caretakers who provide psychosocial character building and socialization skills (through positive communication):

Rules regulating the conduct of both staff and children: (*ASK FOR COPY*)

62. Do all the children receive education and/or vocational training: (*ASK FOR COPY OF EDUCATION CURRICULUM & DAILY SCHEDULES IN THE CHILDREN`S HOME*)

63. Are there regular interaction between the children and the community?

64. Does discrimination of certain groups occur within the children`s home?

65. Is there special focus on **health and nutritional healthcare** for both the girl and the boy child? Yes No Comments: (*OBSERVATION*)

Is there special focus on underweight among adolescent girls?

Is there special focus on vitamin A deficiency among adolescent girls?

Is there special focus on anemia among adolescent girls?

Are the level of malnutrition and disease among girls and boys similar?

Are procedures developed for the early identification of child malnutrition? (*COPY*)

Are all caretakers adequately trained in responses to the discovering of that a child is not eating or drinking enough?

Do both girls and boys receive information on sexuality and family planning?

Acceptability;

66. Does the children`s home provide adequate indoor space to accommodate all children and caretakers: Yes No Comments: (*OBSERVATION*)

Separate bedrooms for boys and girls:

67. Has there been cases of physical, psychological and sexual harassment in the children`s home:

How are adolescent girls and younger children protected?

Affordability;

68. What percentage of the total budget is reserved for healthcare, care and protection?
69. Does access to care and protection services compromise the ability to pay other essential necessities such as food, housing, etc.? Yes No Comment:

Conclusion;

70. The children`s home has sufficient adequate protection and care services, including healthcare services: Yes No Comments:
